# Exploration of the role of emotional expression of treatment-resistant schizophrenia patients having followed virtual reality therapy: a content analysis

**DOI:** 10.1186/s12888-023-04861-2

**Published:** 2023-06-12

**Authors:** Alexandre Hudon, Veronica Lammatteo, Sophie Rodrigues-Coutlée, Laura Dellazizzo, Sabrina Giguère, Kingsada Phraxayavong, Stéphane Potvin, Alexandre Dumais

**Affiliations:** 1 Centre de recherche de l’Institut Universitaire en Santé Mentale de Montréal, Montreal, Canada; 2 Services et Recherches Psychiatriques AD, Montreal, QC Canada; 3 Department of Psychiatry and Addictology, Faculty of Medicine, Université de Montréal, Montreal, QC Canada; 4 Institut national de psychiatrie légale Philippe-Pinel, Montreal, Canada; 5 Université de Montréal, Montréal, Qc Canada

**Keywords:** Psychotherapy, Virtual reality therapy, Auditory hallucinations, Schizophrenia, Avatar Therapy, Emotions

## Abstract

**Background:**

Emotional responses are an important component of psychotherapeutic processes. Avatar therapy (AT) is a virtual reality-based therapy currently being developed and studied for patients suffering from treatment resistant schizophrenia. Considering the importance of identifying emotions in therapeutical processes and their impact on the therapeutic outcome, an exploration of such emotions is needed.

**Methods:**

The aim of this study is to identify the underlying emotions at the core of the patient-Avatar interaction during AT by content analysis of immersive sessions transcripts and audio recordings. A content analysis of AT transcripts and audio recordings using iterative categorization was conducted for 16 patients suffering from TRS who underwent AT between 2017 and 2022 (128 transcripts and 128 audio recordings). An iterative categorization technique was conducted to identify the different emotions expressed by the patient and the Avatar during the immersive sessions.

**Results:**

The following emotions were identified in this study: Anger, Contempt/ Disgust, Fear, Sadness, Shame/ Embarrassment, Interest, Surprise, Joy and Neutral. Patients expressed mostly neutral, joy and anger emotions whereas the Avatar expressed predominantly interest, disgust/contempt, and neutral emotions.

**Conclusions:**

This study portrays a first qualitative insight on the emotions that are expressed in AT and serves as a steppingstone for further investigation in the role of emotions in the therapeutic outcomes of AT.

## Introduction

Schizophrenia is a chronic and complex mental disorder [1]. Despite its low prevalence of around 1% in the general population, it accounts for an annual societal cost of more than the annual cost of all cancers combined and the societal financial cost for care is directly linked to the severity of the disease [2–4]. This psychiatric illness is characterized by the presence of positive symptoms (i.e.: delusions, hallucinations) and negative symptoms (i.e.: alogia, avolition, blunted affect, asociality and anhedonia) [5, 6]. Positive symptoms of schizophrenia are hypothesized to be linked to an increased subcortical release of dopamine, especially in the mesolimbic region (i.e. cortical pathway involving the nucleus accumbens) [7–9]. This results in an increased activity of the dopaminergic receptors D2 and manifests as hallucinations and delusions [10]. It is hypothesized that the negative symptoms can either be intrinsic to the pathophysiology of schizophrenia or can be secondary symptoms that are related to various factors such as adverse effects of treatment, the environment and comorbidities [11]. Functional neuroimaging studies also support the evidence of fronto-temporal dysconnectivity in patients suffering from schizophrenia with several frontal lobe and temporal lobe abnormalities that could yield explanations for positive and negative symptoms [12, 13].

Various antipsychotic pharmaceutical approaches for positive symptoms such as hallucinations are available as first line of treatment [14, 15]. Anti-dopaminergic medication such as dopamine receptor antagonists (i.e. Risperidone, Quetiapine) and partial dopamine receptor agonists (i.e. Aripiprazole, Brexipiprazole) can be used [16]. However, around 30% of patients suffering from schizophrenia are said to be treatment resistant as they either fail to respond or only partially respond to two or more antipsychotic medications [17]. These patients tend to have poorer premorbid social functioning and represent a greater societal financial burden [18]. For these patients, Clozapine is currently the next line recommended pharmaceutical approach, but up to 60% of the patients on this medication will not respond favourably to treatment [19–21]. For these reasons, various adjunct approaches such as psychological therapies have been developed across the years. The main psychological intervention used for patients with treatment resistant schizophrenia is psychosis oriented cognitive-behavioral therapy (CBT) [22]. While CBT has been proven effective for reducing positive symptoms for these patients, the results remain sub-optimal and other strategies have been developed to address this limitation [23, 24].

Amongst these other strategies are virtual reality-based therapies (VRT) such as Avatar Therapy (AT). Developed by Julian Leff and his team in 2008, this psychotherapeutic approach involves the use of an immersive virtual reality system in which patients suffering from treatment resistant schizophrenia (TRS) interact with the Avatar, a virtual representation of their main persistent auditory verbal hallucination which is controlled and animated by the therapist [25]. Several studies are reporting the effectiveness of AT in the reduction of auditory and verbal hallucinations [26–28]. At the *Institut universitaire en santé mentale de Montréal* (IUSMM), AT is a protocolized therapy which is currently being studied with an undergoing trial to compare its effectiveness to CBT. It is designed as a therapeutic process that includes nine therapeutic sessions. The patients attend one AT session per week until completion of the sessions. In the first session the Avatar is being created by the therapist in collaboration with the patient, using a 3D software, to best represent their own representation of their most distressing verbal hallucination. A broad array of features can be employed (gender, facial characteristics, width, height) to design the Avatar. In the remaining eight sessions, the patients will meet and interact with the Avatar using a virtual reality headset. The Avatar is animated by the therapist and the voice of the therapist is modulated using an external voice modifier system to best represent the verbal hallucinations heard by the patient. Facial expressions of the Avatar can be modified in real-time by the therapist by using programmed dimers to modify facial features. The Avatar (including its voice) is therefore personalized for every patient.

While this technique is still being studied and developed, qualitative explorations of the therapeutic processes have been conducted to better understand the intrinsic processes linked to the improvements of patients suffering from TRS undergoing AT. Several themes related to the exchanges between the patient and the Avatar have been elicited and described, as well as the ability to automatically and adequately classify interactions such as self perceptions, beliefs about the voices and emotional responses to them [29, 30]. However, for the latter, little is known in current scientific literature regarding the expression of emotions by the patient and by the Avatar during the immersive AT sessions.

Emotional expression is crucial to the therapeutic process as it enables empathic abilities [31]. In addition to emotion attention and clarity, the integration of emotion regulation training to various CBT approaches has been associated with improvement of psychiatric and medical conditions such as persistent physical symptoms and social anxiety [32, 33].

Despite the blunted affect often portrayed by patients suffering from schizophrenia, they do experience a wide range of emotions; however, clinical access and assessment by the therapist represents a challenging and limiting factor [34]. Acoustic and vocal cues can be useful tools in the evaluation of expressed emotions [37]. Vocal cues and variation in audio samples have been studied and employed in the detection of caricatural emotions to assess them in patients with reduced affects or in patients coming from various cultural backgrounds where emotions can be expressed differently [34, 35].

Considering the importance of identifying emotions in therapeutical processes and their impact on the therapeutic outcome, an exploration of such emotions is needed. The understanding of patient’s emotions as well as the ones expressed by the Avatar in AT to further comprehend the underlying intrinsic therapeutic processes could benefit the outcome of the therapy and ultimately the patient. The aim of this study is to identify the underlying emotions at the core of the patient-Avatar interaction during AT by human-driven qualitative content analysis of immersive sessions transcripts and audio recordings. It is hypothesized that various emotions are experienced throughout the therapeutic process and that those experienced by the patients are often different than those expressed by the Avatar. To our knowledge, no study has yet explored the aspect and dynamic of emotions during AT.

## Methods

### Participants and sampling

Participant data used in this study originates from two completed pilot trials at the Centre de recherche de l’Institut universitaire en santé mentale de Montréal (CR-IUSMM) and one ongoing trial comparing AT to CBT [29, 36]. The data fromsixteen randomly selected participants belonging to the clinical trials registered on Clinicaltrials.gov (identifier number: NCT03585127 and NCT04054778) were used. The participants included in this study were all patients selected based on the same inclusion and exclusions criteria. The inclusion criteria for this study was that the were all patients at the IUSMM, above 18 years of age, and suffering from TRS as defined by the absence of response to two or more dopaminergic antagonists. Furthermore, they all received AT between 2017 and 2022. Each patient participated in 9 psychotherapeutic sessions, each lasting one hour, of which 8 were immersive sessions in which they actively interacted with a virtual representation (the Avatar) of their auditory verbal hallucinations. The first therapeutic session is dedicated to the creation of the Avatar and was not included in the present analysis considering it does not content an immersion component. This represented a total of 128 audio recordings and transcripts. The study has been approved by the ethics committee of CR-IUSMM as part of the protocol for AT.

### Data collection

All of the immersive sessions were recorded and transcribed (audio file and transcripts) by research auxiliaries. Transcripts were then counter-verified by Alexandre Hudon (AH) as per the audio recordings to ensure integrity. The auxiliaries were given a coding guideline which included specific rules to preserve the nature of the therapeutic sessions. Several elements of the coding guidelines are found in Mergenthaler and Stinson ‘s *Psychotherapy Transcription Standards* (1992) [37]. As part of the transcription rules followed by the auxiliaries, verbal utterances and paraverbal utterances were transcribed. Punctuation markers were employed to discriminate between completion of a thought and broken thoughts. Formal and structural aspects included a clear transcript heading, speaker codes and capitalization. Time recordings for each individual interactions and pauses were not part of the coding guidelines.

### Data analysis

A content analysis technique using iterative categorization was conducted, as explained subsequently, to identify the different emotions expressed by the patient and the Avatar during the immersive sessions until saturation of the data [38]. Sophie Rodrigues-Coutlée (SCR), Veronica Iammateo (VI) and AH listened to the audio recordings while reading the transcript interactions to identify emotions as per the content, verbal and audio cues defined in Table 1. These emotions were selected as per Paul Ekman’s and Caroll Izard’s emotion-based theories [39, 40]. Turn of speech was used as the scoring unit.

**Table 1:** Emotions classification as per content, verbal and audio cues

Emotions	Cues
Anger	Irregular or fast speech with an attempt to stop individual violation or preserve dignity [41, 42].
Contempt	Humiliation, attempt to belittle or achieve superiority. Often with lack of respect or cruelty. Sarcasm, provocation, one-sided humor and hostility often seen [41].
Disgust	Involuntary repulsion [41].
Fear	Superficial and rapid breathing, accelerated speech, incomplete declarations, nervous laugher, difficulty to express themselves [41, 42].
Sadness	Slower or irregular speech, monotone, increases in pauses or duration of syllable pronunciation. Tendency to use a larger number of possessive pronouns [42–44].
Shame	Feeling of uselessness, powerlessness, negative self-evaluation [41].
Embarrassment	Inhibition of the capacity to do or say their thoughts, insecurity [41].
Interest	Open-ended questions, validations, respectful engagement in the discussion [41].
Surprise	Audible panting, brief emotion, fast and spontaneous [41].
Joy	Augmentation of the speed pressure, satisfaction, exclamation, excitement, laugher [42].
Neutral	Interactions with no speech or content valence [41]. Everything that cannot be identified as one of the above emotions are included in this category.

The first step conducted in the analysis was to annotate transcripts from AT sessions with the goal of associating the emotions expressed by the patient and the Avatar (as per Table 1) to their dialogue and interactions. An initial round of annotation was done on transcripts coming from 2 randomly selected patients amongst the participants (16 transcripts and 16 audio recordings). This was done using *Qualitative Data Analysis Miner software* [45]. The annotations conducted by VI, SCR and AH were compared by assessing the interrater agreement of the coding of the transcripts. A Scott’s Pi was employed to determine the consensus of the coding conducted by the coders [46]. The list of emotions was restructured, and the categories were updated in relation to the difference and observations found across the coders. This iterative process was repeated until the Scott’s Pi obtained was deemed acceptable and data saturation was achieved. Acceptability was defined as per the SAGE Research Methods: a Scott’s Pi of 0.81–1.00 is indicative of an almost perfect agreement, 0.61–0.80 of a substantial agreement, 0.41–0.60 of a moderate agreement, 0.21–0.40 of a fair agreement, 0.0–0.20 of a slight agreement and less than 0 as a poor agreement [46]. The first iteration of annotations yielded a Scott’s Pi of 0.48, with the main difficulty being distinguishing between shame and embarrassment from audio recordings. Thus, these two categories were merged. The second iteration yielded a Scott’s Pi of 0.51; disgust and contempt were then merged as part of this iteration. Finally, the third iteration yielded a Scott’s Pi of 0.72. A total of 48 transcripts were thus annotated as part of this analysis (3 iterations). Following the third iteration, transcripts for 16 participants were annotated by AH using the final coding grid.

## Results

### Sample characteristics

A total of 128 transcripts and audio recordings were analyzed from 16 participants that had received AT. The sociodemographic characteristics of these participants can be found in Table 2.

**Table 2:** Summary of participants’ characteristics

Characteristics	Value (N = 16)
Sex (male, female)	13,3
Age (mean in years)	40.8 ± 9.4
Education (mean in years)	12.8 ± 3.0
Ethnicity (Caucasian, others)	93.8%,6.2%
% on Clozapine	37.5%

Nine emotions were identified across the transcripts. A final description of these emotions can be found in Table 3.

**Table 3:** Redefined coding grid as per the iterative process

Emotions	Cues
Anger	The interlocutor presented an irregular or a faster response than usual with a clear attempt to preserve their dignity or cease an individual violation of their integrity.
Contempt/ Disgust	The interlocutor uses humiliation, belittlement or lack respect to their interlocutor. They may use sarcasm, provocation, hostility, or sound repulsed.
Fear	The interlocutor demonstrates difficulty in completing declarations, laugh nervously, portray difficulty to express themselves.
Sadness	The interlocutor adopts a slower speech with a monotone tonality. There is a clear increase in pauses or an increase in the use of possessive singular pronouns.
Shame/ Embarrassment	The interlocutor’s speech content depicts uselessness, powerlessness, and a negative perception of Self.
Interest	The interlocutor uses open-ended questions, shows interest to what their peer is saying and engages respectfully in the discussion.
Surprise	The interlocutor has a spontaneous response that displays a brief emotion, with an acceleration of speech and audible panting.
Joy	The interlocutor demonstrates a clear increase in satisfaction, excitement, laughs and has an increase in the speed of their speech.
Neutral	The interlocutor’s interaction has no speech or content valence such as a change in tonality, in speed or engages conversation with neutral content.

### Emotions

#### Anger

Anger was identified in the verbalizations and responses of most of the patients and almost never expressed by the Avatar. Anger was mostly represented by an increase in patient tone and associated with an attempt to preserve their dignity. The anger was principally aimed towards the Avatar. An example of a patient expressing anger towards the Avatar is as follows:

*Patient 018: “I’m fed up with our conversations. I’d like you to leave, forever and that you stop threatening me everyday. Please leave now.”*

Another source of anger is linked to the patient’s emotional attachment to the Avatar.

*Patient 2020: “This is exactly why I do not like you and will never love you. It has been 20 years: you never showed any empathy towards me.”*

The Avatar expresses anger solely when there is a clear provocation linked to its existence.

*Avatar 2020: “But a small while ago you said I was an illness, and now you say that I do not exist? I do not follow!”*

Or another clear example is in transcript 001 – T3:

*Patient 001: “I hate you, go die!”*

*Avatar 001: “No, I hate you more! You go die!”*

#### Contempt and disgust

Contempt and disgust are both emotions that were not equally expressed by patients and Avatar; more specifically, the patients rarely demonstrated these emotions across the immersive sessions whereas the Avatar consistently expressed them.

Patients expressed contempt and disgust mainly when confronted to a statement made by the Avatar that goes against their values or their views on a particular subject.

Example from transcript 041 – T6 :

*Avatar 041: “I would love to spend much more time with you.”*

*Patient 041: “I do not feel that way at all.”*

As per the Avatar, attempts to elicit reactions from the patients appear to be what drives the expression of contempt and disgust.

*Avatar 006: “I feel it deep inside you that you have no self-esteem, which is why I can stay as long as I want in your head.”*

#### Fear

Fear has been expressed by both the patients and the Avatar throughout the transcripts in a consistent fashion. Patients’ fear was mostly characterized by the difficulty in completing their sentences whereas the Avatar expresses fear in an exaggerated manner to empower the patient.

The participants manifested fear principally when interferences with primary needs were brought up or threatened during conversation.

Example from 039 – T7:

*Avatar 039: “I can’t wait for you to be in the streets.”*

*Patient 039: “In the street I will not be able to have my medication because I will not have a fixed address.”*

#### Sadness

Sadness was experienced summarily by the patients and the Avatar towards the end of the AT. In both cases, it was either in relation to belittlement, patient-Avatar affiliation or when the patient verbalized negatively valanced thoughts and claims concerning the Avatar.

Example in transcript 1041 – T8.

*Avatar 001: “How come you don’t like it that much?”*

*Patient 001: “I don’t like my workplace, I don’t like my colleagues.”*

Separation elicited by the patient towards the Avatar also yielded an expression of sadness from the Avatar.

An example can be found in transcript 001-T8:

*Patient 001: “You will not be in my life anymore; you will be in a calm space.”*

*Avatar 001: “But, but I want to be with you! It’s been 40 years.”*

#### Shame and embarrassment

The expression of shame and embarrassment was very rarely seen by the Avatar as compared to the patients. Themes and ideas of de-valorisation (negative perception of self) were common amongst all the patients’ transcripts. They were mostly consequent to the use of provocation or belittlement from the Avatar. As per the Avatar, the expression of shame was solely seen when the patient used self-empowerment to respond to the mere existence of the Avatar:

*Patient 001: “As of now, I will not fear the words coming out of you. You are not reliable.”*

*Avatar 001: “I feel very small.”*

#### Interest

Throughout the transcripts, it can be observed that the Avatar expresses interest towards the patients whereas the patients do not seem very interested in asking the Avatar open-ended questions.

During AT sessions, the Avatar attempts to elicit responses from the patients. One strategy often employed is the use of open-ended questions, which favours access to the patient’s point of view on a specific subject.

As an example, in transcript 2020 – T5:

*Avatar 2020: “And why do you think we could not live together?”*

*Patient 2020: “Because I try to do what my doctors told me to do.”*

Interest is expressed by the patients mainly when they try to obtain the point of view of the Avatar in connection with an action conducted by their auditory hallucinations.

Example from transcript 1039 – T4:

*Patient 1039: “I’d like to know why you come to visit me in the evening.”*

*Avatar 1039: “I visit you to make fun of you.”*

#### Surprise

The emotion of surprise was anecdotical as per patients’ expressions whereas it was a common expression identified from the Avatar. Though rare, this emotion was identified in patient’s responses when they were challenging the Avatar and the Avatar responded with a validating or positively valanced open-ended question.

An example of such interaction is identified in transcript 006-T4:

*Patient 006: ‘‘Well, you are not speaking to me since a few days.”*

*Avatar 006: “How did you succeed in making me the quiet one?’‘*.

*Patient 006: “how did I succeed?”*

The Avatar portrayed more exaggerated surprised responses when challenged by the patient on various topics related to their social functioning or their hallucinations.

This is well illustrated in transcript 018 – T4:

*Patient 018: “The voices they come from my illness.”*

*Avatar 018: “What illness?”*

*Patient 018: “Schizophrenia.”*

*Avatar 018: “Are you saying that I am a disease!!?”*

#### Joy

Joy was almost never seen as an expressed emotions from the Avatar throughout the transcripts whereas it was one of the most common emotions from the patients, especially towards the end of immersive sessions.

Patient’s expressed joy when they appeared to be in control in relation to the provocation conducted by the Avatar.

As an example, in transcript 1041 – T7:

*Avatar 1041: “I told you that you would find it hard without me.”*

*Patient 1041: “Oh but no worries, I will be fine! Time will act against you.”*

As for the Avatar, joy was expressed solely to try and elicit further reaction from the patient when faced with a closed-ended affirmation.

In transcript 018- T4 demonstrates such interaction:

*Avatar 018: “Me, I love to have power.”*

*Patient 018: “I have no doubt about this.”*

*Avatar 018: “And I’m so happy because you gave me this power.”*

#### Neutral

Neutral verbalizations were the most popular ones observed amongst the patients. Normal tone with no particular associated content constitute the type of interactions that were encountered, especially at the beginning of the immersive sessions.

These interactions where also linked to the technical aspects of the immersive sessions in which the Avatar might validate certain specificities such as display brightness.

For example, in transcript 1039- T5:

*Avatar 1039: “Do you see me?”*

*Patient 1039: “Yes. I see you.”*

## Discussion

The objective of this study was to explore the emotions of patients’ suffering of TRS and that have undergone AT. It was also designed to identify the emotions expressed by the Avatar throughout the immersive sessions. Nine emotions were identified across the transcripts: Anger, Contempt/ Disgust, Fear, Sadness, Shame/ Embarrassment, Interest, Surprise, Joy and Neutral. Neutral, joy and anger were the emotions that were mostly expressed by the patients. As for the Avatar, expression of interest, disgust/contempt and neutral were amongst the emotions the most annotated across the transcripts.

Patients and Avatars in this study expressed various emotions during the psychotherapeutic process of AT. A recent thematic qualitative evaluation of AT involving views of 15 patients on the therapeutic process identified voice embodiment and associated emotions as a major theme, considering that the voice of the Avatar triggers emotional responses [47]. This can explain why various emotional responses were identified in the presented study and why there seems to be different links between specific verbalizations by the Avatar and the emotional response expressed by the patient. Another recent study explored emotion elicitation in virtual reality for 11 participants and demonstrated the possibility to elicit fear and anger in a secured immersive environment [48]. Similarly, virtual environments themselves have been found to intrinsically elicit emotions across patients in virtual reality settings [49].

While similar studies are not identified for patients suffering from TRS, the polarity between anger and joy, the most frequently identified emotions across the transcripts for the patients (after neutral emotions), might be explained by the neurophysiological changes observed in patients suffering from TRS. Neuroimaging studies have reported that emotionally laden images elicited hyper-activation in the dorso-medial prefrontal context and left cerebellum in TRS patients [50]. In another study, weaker cerebellum activity presented with deficits in emotion recognitions in schizophrenia [51]. Since the Avatar is visually represented and presents modifications of facial expressions, this might trigger these hyper-activations and oscillations between emotional responses of anger, neutral versus joy, rarely including the other emotions. It is also important to note that emotion identification is possible in patients suffering from schizophrenia and although their affect might not display their emotional processes and responses, they can feel them [52]. As for the fear response in the patients, it has been found that virtual avatar and human responses can both elicit the same response in the amygdala even if the avatar are overly anthropomorphic [53]. Further understanding of these emotional responses could be elicited using a wider array of parameters such as heart rate, body temperature, gesture, and overall behavior.

As for the Avatar, as part of the therapeutical processes, it is important for the therapist to elicit patient reactions. Interest, similarly, to most positive emotions, is an emotion that can be strategically employed to create the therapeutic alliance and to reduce the anxiety and fear linked to the therapeutic sessions themselves [54, 55]. It is also an emotion that can help the therapist to use real and personalized examples to confront or validate the patient, which may explain why interest is such a frequently annotated emotion throughout Avatar transcripts. The emotional expressiveness is an important component of the therapy and it has been demonstrated that simulation of Avatars through virtual reality is a way to train patients suffering from schizophrenia in their abilities to recognize certain emotions [56].

### Limitations

This is an exploratory study and the lack of generalisation of the results can limit its interpretation. Considering the emotional responses, the emotion identification process could have been biased by the coders’ own understanding and perception of emotions. This has been mitigated using an emotional grid that was defined and the interrater analysis. This study does not include the patient’s own labelling and identification of his or her emotions underlying their reactions and transcripts and there could thus be a mismatch between the coders’ perceptions and patients’ perceptions of their own emotions. Another limitation is that visual cues were not taken into consideration which limits the analysis to the content of the verbatims and the audio transcripts. The random selection of the participants could yield to bias considering that emotionally tinged speech can vary for men and women [57].

## Conclusion

To conclude, the main objective of this study was to explore the emotions of TRS patients and the Avatar in AT. The use of an iterative categorization content analysis process enabled the identification of nine emotions. The nine emotions were identified across the transcripts and appeared to be linked with Avatar-Patient dynamics as particular emotional responses were often seen in contexts of provocation or belittlement. Emotional expressions were more polarized in the patients where anger, joy and neutrality were predominant as compared to the Avatar where interest, disgust/contempt and neutrality were more observed. While this study portrays a first qualitative insight on the emotions that are expressed in AT, further studies are needed to assess their role in the treatment response associated to the immersive session of AT.

## Data Availability

The datasets generated and/or analysed during the current study are not publicly available due to patients’ privacy but are available from the corresponding author on reasonable request.

## List of Abbreviations

AT: Avatar Therapy
CBT: Cognitive-behavioral therapy
TRS: Treatment resistant schizophrenia
